# Complex couplings between joints, muscles and performance: the role of the wrist in grasping

**DOI:** 10.1038/s41598-019-55443-w

**Published:** 2019-12-18

**Authors:** Mathieu Caumes, Benjamin Goislard de Monsabert, Hugo Hauraix, Eric Berton, Laurent Vigouroux

**Affiliations:** Institute of Movement Sciences, National Centre for Scientific Research, Aix-Marseille University, Marseille, 13009 France

**Keywords:** Computational models, Motor control, Biomedical engineering, Mechanical engineering

## Abstract

The relationship between posture, muscle length properties and performance remains unclear, because of a lack of quantitative data. Studies on grasping tasks suggested that wrist position could favour the extrinsic finger flexor in regards to their length to maximise grip force performance. The present study aimed at providing quantitative evidence of the links between wrist posture, muscle capacities and grip capabilities. It combines experimental measurements and a musculoskeletal model including the force-length relationship of the four prime muscles used in grasping. Participants exerted their maximum grip force on a cylindrical dynamometer in four different wrist postures, including one freely chosen by participants (spontaneous). A musculoskeletal model computed the muscle force level and length from motion capture and muscle activation. Results revealed that participants exerted maximum grip force spontaneously, with a loss of force when using other postures. At muscle force and length level, grip force variation seems to be associated with all the muscles under study. This observation led to a first quantitative link between power grip, posture and muscle properties, which could provide more insight into neuromechanical interaction involved when grasping. The design of ergonomic devices could also benefit from this quantification of the relationship between wrist angle and muscle length properties.

## Introduction

Prehension is a crucial capability for our day-to-day activities. It allows us to manipulate a vast range of objects of different sizes and shapes during numerous tasks that may require both precision and/or forceful exertion. Although we perform grasping tasks naturally, such action requires the coordination of the multiple degrees of freedom of the fingers and the wrist, and the numerous muscles of the forearm and the palm to meet both the mechanical constraints (e.g. object stability and force level), and the task goal (e.g. hold, throw, write, etc). Furthermore, the specific anatomical configuration of the hand involves strong coupling between the wrist and the finger joints, since several finger muscles, called extrinsic, originate in the forearm and insert via long tendons on distal phalanges. During grasping tasks, these muscles are solicited to exert forces on the object and concomitantly create action at the wrist, meaning that gripping actions are inherently linked to the mechanical equilibrium of the wrist^1,2^. This illustrates the inherent couplings between task constraints - here the force level - and biomechanical properties - here the wrist equilibrium. Although the existence of this biomechanical functioning has been known for many years, the way such wrist-finger muscular interactions influence the control of grasping is still not fully understood. Some studies have reported stereotypical movement in reach to grasp task^3,4^ and typing^5^ even if a large number of joints is involved. These results suggest that there is a limited number of joint postures for successfully grasping an object. For the finger postures, this behaviour is understandable, since the shape and size of an object will constrain the finger joints. On the contrary for the wrist joint, it is surprising that participants spontaneously choose a specific wrist position^6^ when exerting a maximum grip force while the wrist can potentially move freely in a wide range of positions. More interestingly, this spontaneous posture resulted in the highest grip force compared with other positions. Knowing that the extrinsic flexor muscles simultaneously produce the grip force and act at the wrist, it has been suggested that this spontaneous wrist position corresponds to a more advantageous configuration for such muscles. Hence, the posture that allows the best force capabilities may emerge spontaneously from anatomical properties to satisfy both the mechanical constraints and the goal of the task. This hypothesis is particularly interesting as it suggests a direct link between the local constraints of the neuromusculoskeletal system, dictated by joint and muscle couplings, and the task requirements. Unfortunately, because of a lack of quantitative information on the way the wrist posture influences muscle force capacities, the hypothesis has never been confirmed.

Several studies have evaluated the interactions between wrist posture and hand force production during pinch grip tasks^7,8^ or power grip tasks^6,9,10^. Overall, studies have shown an effect of wrist position with variation characterised by a loss of grip force in extension and flexion compared with more neutral positions, i.e. 0 degrees of flexion and deviation. For pinch grip, around 10 to 20% of grip force is lost in extension and 20 to 30% in flexion^7^. For power grip tasks, studies showed that the maximum grip force could decrease by 30% when flexing and 10% when extending the wrist from the spontaneously taken optimum position. The hypothesis advanced for these losses were associated with the decrease in the force-generating capacities of the extrinsic finger flexors, i.e. the main agonist of force exertion in grasping^10–12^. The force which a muscle can produce is indeed dependent on its length, via the well-known force-length relationship^13–15^. This relationship presents an optimum length that provides a maximum level of force and is such that, the further away from this optimum length, the lower the level of force the muscle can achieve. As the position of the wrist influences the current length of the extrinsic finger muscles, the optimum wrist position could indeed correspond to an optimum point in terms of muscle capacities. However, because little information is available regarding the relationship between wrist posture and extrinsic muscle length as well as the force-length behaviour of hand muscles, this hypothesis has never been verified. Because the direct *in vivo* measurement of muscle length and force is invasive, musculoskeletal models have been used to assess such variables. These models mostly rely on relatively meagre data taken from a corpse^16–18^ which can lead to imprecision in length estimation and thus the force-length modelling in positions other than those examined in previous studies. A recent study^19^ has however characterised the *in vivo* muscle force-generating capacities of representative muscles used for grasping according to the length variations using ultrasound protocols. These relationships allow us to explore the influence of joint postures on muscle force-length properties and associate its variations with a performance, here the force level in power grip.

The present study aims to analyse the influence of wrist posture on both grip force capabilities and muscle force-generating capacities and provide a rationale for the hypothesis concerning the spontaneous wrist posture, discussed above. A protocol was developed to measure grip force, hand kinematics and muscle activity in different wrist positions during maximum force exertion on a cylindrical dynamometer. A musculoskeletal model of the index finger and wrist was developed to estimate the current length and force of four representative muscles according to their activities and hand joint positions. We hypothesise that (i), maximum grip force will occur for a spontaneous wrist posture in comparison with imposed postures, (ii) the four muscles will be affected differently by wrist postures in terms of force-generating capacities and (iii) the variations of maximum grip force induced by the wrist posture cannot be fully explained solely by the force-generating capacities of extrinsic finger flexors.

## Results

The influence of wrist posture on grip capabilities and muscle-force generating capacities was evaluated by combining an experimental protocol and a musculoskeletal model of the hand. Regarding the experimental task, participants were instructed to exert maximum grip forces on a cylindrical handle with three diameters and four postures. One posture was freely chosen by the participants, referred to as the spontaneous position, while the three others were imposed on the participants (flexion, neutral and extension). The grip force exerted was recorded with an instrumented handle simultaneously with the kinematics of hand and index finger, using motion capture, and surface electromyographic signals of four extrinsic muscles (see Methods Section). The muscles investigated were: the index finger flexor digitorum superficialis (FDS) and extensor digitorum communis (EDC) and the flexor carpi radialis (FCR) and extensor carpi radialis (ECR). From these data, the current length and force of each muscle were estimated using a musculoskeletal model of the wrist and index finger that consisted of two steps. First, the model estimated the musculo-tendon excursion of each muscle from kinematics. Secondly, from the activation level and musculo-tendon excursion, the length and force of each muscle were evaluated through polynomial equations describing force-length-activation relationships determined from *in vivo* data in a previous study^19^. From this muscle length and force data, regression models were tested in order to determine the best variable (force or length) and the combination of muscles that could explain grip force variations.

The results of the experiment showed that the spontaneous wrist posture was reproducible and similar across participants and resulted in the highest maximum grip forces. Remarkably, when considering the results at the muscle levels, finger flexor was the least affected by wrist angle changes, compared with other muscles in terms of both force levels and lengthening, which contradict the general hypothesis of a direct link between grip force variation and finger flexor length. Furthermore, results from regression tend to show that grip force variations are explained by a combination of all the muscles involved, with ECR playing a significant role. This result opens possibilities of exploring new criteria, based on muscle capacities, on how the neuromusculoskeletal model chooses joint postures to maximise the performance in a task

### Experimental data

#### Wrist angle

**Figure 1:**
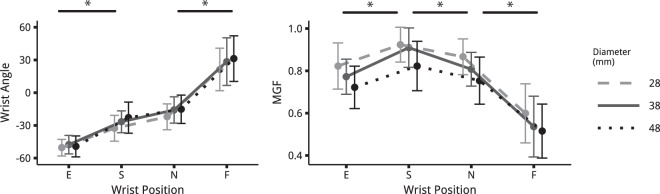
(**a**) Mean ± one standard deviation values of the measured wrist angle taken by the participants at each *diameter* against the *posture* conditions, i.e. extension (E), spontaneous (S), neutral (N) and flexion (F). (**b**). Mean ± one standard deviation values of the normalised maximum grip force (MGF) for each *diameter* against the *posture* conditions.

The results of the wrist flexion-extension angles taken by the participants for each *posture* - i.e. extension (E), spontaneous (S), neutral (N) and flexion (F) - and *diameter* - i.e. 28 (D28), 38 (D38) and 48 mm (D48) - are shown in Fig. 1. The values varied from −49.1 ± 8.62 degrees for extension (E) on D48 to 27.0 ± 21.1 degrees for flexion (F) on D28. Statistical analysis showed a significant effect of *posture* (*F*(3,45) = 162.00; *p* = 1.03 · 10^−14^) and a significant effect of *diameter* (*F*(2,30) = 13.55; *p* = 6.41 · 10^−5^) on the wrist flexion-extension angle. No interaction of *posture* × *diameter* was found on wrist angle (*F*(6,90) = 1.25; *p* = 0.29). From the extension (E) posture to the flexion (F) one, the angle increased progressively with each posture being significantly different from the others (Tukey HSD *pvalue* < 0.0001 for each pair) except for the spontaneous (S) vs neutral (N) posture (*p* = 0.044). The only significant difference observed regarding the *diameter* effect was that the smaller one, i.e. D28 resulted in smaller wrist angles compared with the larger ones.

#### Normalised maximum grip force

The results concerning the maximum grip force reached by the participants are plotted in Fig. 1. The highest MGF was produced for the spontaneous (S) posture and D28, resulting in a mean value of 0.924 ± 0.08, and the lowest level was for the flexion (F) posture and D48, corresponding to a mean value of 0.515 ± 0.128. The two main factors had significant effect on the maximum grip force (*F*(3, 45) = 82.98; *p* = 6.65 · 10^−12^ for *posture*, *F*(2, 30) = 6.96; *p* =3.27 · 10^−3^ for *diameter*. Interaction between *posture* and *diameter* was not significant (*F*(3,45) = 0.78; *p* = 0.59). Concerning the positions, the maximum grip force produced for the spontaneous (S) posture was higher than for the other ones (*p* < 0.007 at most). For the *diameter*, only the maximum grip force produced on D48 differed from D28 (*p* = 0.002) and resulted in lower values.

#### Muscle activation

A significant effect of both *muscle* and *posture* (*F*(3,45) = 12.87; *p* = 3.33 · 10^−6^, *F*(3,45) = 2.91; *p* = 4.49 · 10^−2^) was found on muscle activation but no effect of *diameter* was found (*F*(2,30) = 1.35; *p* = 2.75 · 10^−1^). All interaction effects including the *Muscle* factor, were significant (*Muscle* × *Posture* (*F*(9,135) = 3.78; *p* = 2.81 · 10^−4^), *Muscle* × *Diameter* (*F*(6,90) = 3.31; *p* = 5.41 · 10^−3^), *Muscle* × *Posture* × *Diameter* (*F*(18,270) = 1.81; *p* = 2.29 · 10^−2^)). Interaction of diameter × posture was not significant (*F*(6,90) = 0.64; *p* = 6.92 · 10^−1^). Activation levels of FDS (0.69 ± 0.13) were significantly higher than those of FCR (0.49 ± 0.18) and EDC (0.55 ± 0.14) but not different from those of ECR (0.63 ± 0.14).

### Modelling

#### Muscle length

**Figure 2:**
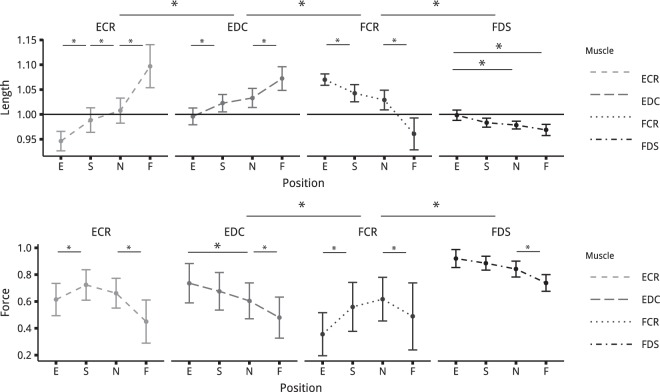
Mean normalised force and length of the four muscles for the different postures. Upper panel presents force values and lower panel length values. *posture* goes as Extension (E), spontaneous (S), neutral (N) and flexion (F). Muscle abbreviations are FCR for *Flexor Carpi Radialis*, ECR for *Extensor Carpi Radialis*, FDS for *Flexor Digitorum Superficialis* and EDC for *Extensor Digitorum Communis*.

The normalised length of each muscle estimated in the different postures from experimental kinematic and EMG data using the musculoskeletal model are presented in Fig. 2. The main effects of *muscle* and *posture* on muscle length were significant (*F*(3,45) = 214; *p* < 1 · 10^−15^, *F*(3,45) = 54; *p* = 6.66 · 10^−15^) and their interaction, *Muscle* × *Posture* was significant (*F*(9,135) = 153; *p* < 1 · 10^−15^). The effect of *diameter* and its interactions with other factors were not significant. (*F*(2,30) = 2; *p* = 1.89 · 10^−1^ for *diameter*, *F*(6,90) = 1; *p* = 4.76 · 10^−1^ for *diameter* × *posture*, *F*(18,270) = 1; *p* = 1.45 · 10^−1^ for *muscle* × *posture* × *diameter*). For FDS, the length in the spontaneous (S) posture was not different from that in other postures. (*p* > 0.31). ECR presents the widest length variations, with a shortest length in extension (E), and a longest in flexion (F) (*p* < 0.038 at most for all combinations) For the 2 other muscles, the only non-significant difference was for the length in the neutral (N) and spontaneous (S) posture.

#### Muscle force

The normalised force of each muscle estimated in the different postures from the normalised length and EMG data using the musculoskeletal model are presented in Fig. 2. Statistical analysis revealed a significant effect of *muscle* and *posture* on muscle force levels (*F*(3,45) = 41.56; *p* = 5.04 · 10^−13^, *F*(3,45) = 4.42; *p* = 2.40 · 10^−8^) and no significant effect from *diameter* (*F*(2,30) = 2.86; *p* = 7.82 · 10^−2^. All interactions were significant (*muscle* × *posture* (*F*(9,135) = 19.052; *p* < 1 · 10^−15^, *muscle* × *diameter* (*F*(6,90) = 4.45; *p* = 5.77 · 10^−4^, *diameter* × *posture* (*F*(6,90) = 2.70; *p* = 1.85 · 10^−2^, *muscle* × *posture* × *diameter* (*F*(18,270) = 2.25; *p* = 2.94 · 10^−3^). When comparing between muscles, the FDS presented the highest force level, and FCR the lowest one (*p* < 0.007 at most for both). No significant differences were found between ECR and EDC force levels (*p* = 0.98). Comparison between postures showed that the force level of FDS was significantly lowest for the flexion (F) posture, and similar among all the other postures. ECR force levels in extension (E) and flexion (F) posture were significant compared with the spontaneous (S) one (*p* < 0.008 at most). There was no significant effect of *posture* on EDC and FCR.

#### Model regression

The results of linear or quadratic regressions with different combinations of either force or muscle length are presented in Table 1. Linear models provided comparable results to quadratics in terms of fitting quality and ranking of muscle combinations but were more restrictive when considering best models. Differences in model ranks between linear and quadratic models were lower when force was the dependent variable compared with those based on muscle length.

**Table 1:** Δ_*AIC*_ and associated rank for each single muscle models, 2 multiple regression models that are coherent with our hypothesis and the model combining different muscles with the lowest Δ_*AIC*_.

		Force						Length					
		Linear			Quadratic			Linear			Quadratic		
		Rank	Δ_*AIC*_	${R}_{adj}^{2}$	Rank	Δ_*AIC*_	${R}_{adj}^{2}$	Rank	Δ_*AIC*_	${R}_{adj}^{2}$	Rank	Δ_*AIC*_	${R}_{adj}^{2}$
Single Muscle Mode	FCR	15	77.99	0.00	15	80.84	0.03	7	9.74	0.26	11	27.83	0.28
	FDS	7	19.66	0.26	8	14.91	0.31	15	50.89	0.09	13	32.46	0.26
	EDC	13	68.91	0.05	14	67.86	0.09	13	40.17	0.13	15	43.54	0.22
	ECR	10	34.92	0.20	11	43.59	0.20	11	13.59	0.25	14	32.61	0.26
Multiple Muscle Models	FDS + ECR	3	2.00	0.33	4	3.50	0.36	12	14.60	0.25	10	22.25	0.31
	FCR + FDS + EDC + ECR	2	1.99	0.34	1	0.00	0.38	4	3.49	0.30	3	3.01	0.39
**Most Plausible**	**Values**	**1**	**0.00**	**0.34**	**1**	**0.00**	**0.38**	**1**	**0.00**	**0.30**	**1**	**0.00**	**0.39**
	**Muscles**	**FDS** + **EDC** + **ECR**			**FCR** + **FDS** + **EDC** + **ECR**			**FCR** + **EDC**			**FDS** + **EDC** + **ECR**		

The single muscle models did not yield plausible results, with the best ones being obtained with FDS when force is the dependent variable (linear: 7th rank, Δ_*AIC*_ = 19.66, *R*^2^ = 0.26 and quadratic: 8th rank, Δ_*AIC*_ = 14.91, *R*^2^ = 0.31) and with FCR when length is the dependent variable (linear: 7th rank, Δ_*AIC*_ = 9.74, *R*^2^ = 0.26 and quadratic: 11th rank, Δ_*AIC*_ = 27.83, *R*^2^ = 0.28). Inversely, the least plausible models (highest Δ_*AIC*_) were obtained with FCR when force was the dependent variable and with FDS when the length was the dependent variable. ECR corresponded to the second most plausible model for both variables (Force/Linear: Δ_*AIC*_ = 34.92; Length/Linear: 13.59).

Multiple muscle models provided more plausible models, i.e. lower Δ_*AIC*_ values. Three models are considered within the 24 possible combinations, FDS + ECR, all-muscles model and the model with Δ_*AIC*_ equal to zero, i.e. the most plausible one. The first one combines the two muscles that are assumed to be the most influential on grip force, and the second one was chosen because it includes all the muscles considered in this study. According to the Akaike Criterion scale (described in Method Section), FDS + ECR is a substantial model when linear force is considered with Δ_*AIC*_ equal to 2 and ${R}_{adj}^{2}=0.33$. It becomes less plausible when force is a quadratic model and becomes implausible when length is considered (Δ_*AIC*_ > 14.60). The all-muscles model is substantial when force is considered (Δ_*AIC*_ < 1.99 and ${R}_{adj}^{2}=0.34$ for linear model and ${R}_{adj}^{2}=0.38$ for quadratic model). It becomes less plausible when length is considered (3.01 < Δ_*AIC*_ < 3.49). Finally, the most plausible model for linear force and quadratic force are FDS + EDC + ECR $({R}_{adj}^{2}=0.34)$ and all muscle model $({R}_{adj}^{2}=0.38)$, respectively. The most plausible models for linear length and quadratic length are FCR + EDC $({R}_{adj}^{2}=0.30)$ and FDS + EDC + ECR $({R}_{adj}^{2}=0.39)$.

## Discussion

This study intended to explore the complex interactions between joint posture, muscle capacities and task performance through the study of a grasping task, which involves hand and wrist couplings. An experimental protocol and a musculoskeletal model were developed to quantitatively characterise the influence of wrist posture on the grip force capabilities and the force-generating capacities of the hand muscles. We have sought to provide a rationale based on the muscles’ physiological state to explain why the wrist posture we spontaneously adopt to exert a maximum grip force corresponds to an optimum case in terms of performance. Studying this wrist-hand coupling is interesting as it could provide a better understanding of the mechanism underlying the emergence of a specific joint configuration - here the wrist position - when facing a particular constraint - here maximising grip force. The analysis at the muscle level we conducted was made possible by the results of a previous study that provided a new modelling of the force-length relationship based on data from *in vivo* experimentation, i.e. ultrasound imaging combined with EMG, kinematic and torque measurement^19^

The results of our protocol confirmed that wrist angle has a critical influence on grip force variations. When asked to grasp a cylindrical handle with maximum force, participants spontaneously adopted a reproducible position which resulted in the highest force produced (Fig. 1). When other wrist postures where imposed, the loss in grip force capability could be close to 30% which is consistent with values available in the literature^6,10,11^. The evolution of the grip force with wrist posture followed an inverted bell-shaped curve, as previously reported in the literature^6,10^. The lower forces observed for the extension and flexion postures can arise from a wrist angle less mechanically favourable for extrinsic muscles and wrist actuators. Indeed, concerning the force-length, when the muscles are far from their optimum position, i.e. flexion or extension in our conditions, they suffer a greater loss of force than when they are closer to the optimum, i.e. spontaneous or neutral. Our protocol intended to observe this effect of posture on grip force in order to answer whether the variations observed can be attributed to changes in muscle force-generating capacities induced by the wrist position.

**Figure 3:**
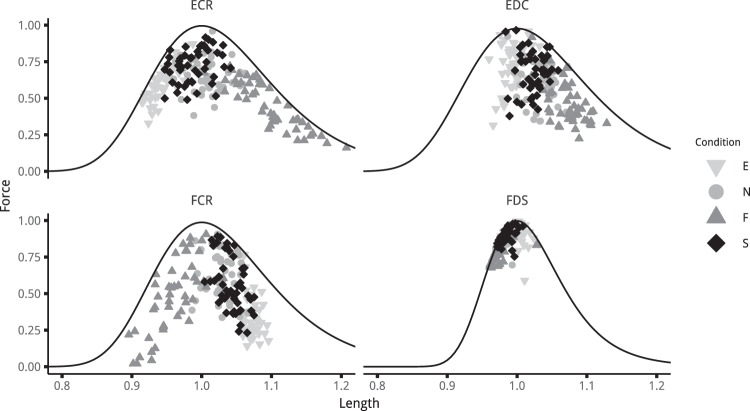
Normalised force against length of the four muscles all participants and all conditions (3 diameters and 4 postures). The black line represents the force-length relationship at activation =1. Muscles are in their optimum length at 1.

The estimations provided by the musculoskeletal model, based on *in vivo* data, confirmed that each muscle is affected differently by wrist posture (Fig. 3). Among all the four muscles investigated in this study, FDS was the least affected by wrist angle variations in terms of force levels and lengthening. Both FDS force and length were close to the optimum, regardless of the wrist posture taken by the participants. This remarkable result challenges the commonly held assumption that grip force variations associated with wrist posture are due to changes in FDS length leading to less advantageous force-generating capacities^10–12^. More surprisingly, the ECR muscle is markedly affected by wrist angle, both in force and length. Furthermore, the variations of ECR force followed a trend similar to the one of the grip force with an optimum force level at the spontaneous posture (S) and an important difference between maximum and minimum values (36% loss). This result could seem surprising since ECR is a wrist extensor muscle, inserting on the second metacarpus, which has no direct action on the fingers. Nevertheless, as found in previous studies, this muscle is necessary for stabilising the flexion moments inherently created by the finger flexors at the wrist joint when they exert forces on the grasped objects^20,21^. Compared with these previous studies, the results of our experiment suggest that ECR force-generating capacities seem to have a direct impact on the grip force level and that the role of the wrist extensors is crucial in the interactions between wrist posture and gripping actions. The two other muscles (FCR and EDC) exhibit less remarkable results but clarify their role in the control of grip force. Just like the ECR muscle, the FCR was markedly affected by wrist posture in terms of both length and force. However, its optimum length was between the neutral and the flexion postures, whereas other muscles reached it around the neutral or extension posture. Moreover, FCR EMG activation was relatively low with great discrepancies across subjects. These two observations seem to follow the assumption that the neuromusculoskeletal system might choose to activate muscles only when their force-generating capacities are optimum^22^. As a result, despite the fact that its force-generating capacities are affected by wrist posture, our results suggest that this muscle plays only a minor role in grip force. Lastly, EDC mostly travels the descending part of the force-length relationship, and was, as FDS, less affected by wrist posture than wrist muscles (ECR and FCR). This can be explained anatomically, with finger actuators being deeper and closer to the wrist’s centre of rotation than wrist actuators. This anatomical configuration implies that the wrist posture has a weaker influence on finger actuators than prime wrist movers. Thanks to this musculoskeletal arrangement, probably resulting from evolution, high gripping actions can be maintained over the wrist range of motion, therefore providing the hand with the ability to ensure a stable grip even when orienting the objects in different positions, e.g. when avoiding obstacles. All these results support the hypothesis that the decrease of grip force observed with extension and flexion of the wrist cannot be associated solely with the changes in extrinsic finger flexors but is rather a complex interaction between all muscles mobilising the wrist.

**Figure 4:**
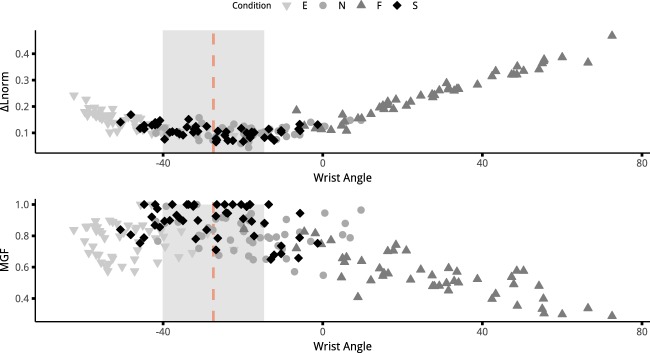
Upper panel presents the sum of the difference between the normalised length of the four muscles and their optimum normalised length against the wrist angle. The data from all participants and all conditions (3 diameters and 4 postures) are plotted. The lower the value, the closer to their optimum are the muscles. The lower panel shows the normalised grip force against the wrist angle. Red dashed line represents the mean spontaneous (S) posture, with its standard deviation represented by the grey area.

A multiple regression analysis was conducted to select the parameters, i.e. combinations of muscles and variables (length or force), that best explain grip force variations. A first conclusion emerging from regression results confirmed that grip force variations were not correlated with either the force or length variations of a single muscle. These factors are very unlikely to reflect grip force variation compared with regression based on a combination of muscles. More specifically, it appeared that the combination of FDS, ECR and EDC seemed to offer a plausible explanation for grip force variations with the combination of all four muscles. The results of the analysis also confirmed that FCR could be correlated with grip variations, if only length was considered, but was generally not appearing in the muscle combinations providing the most plausible model. While grip force variations according to wrist posture are a well-known phenomenon, no studies have yet explored this paradigm at the level of muscle force-generating capacities. Thanks to the result of a recent study providing more accurate modelling of the force length-relationship of hand muscles, we were able to observe that FDS lengthening does not seem to be the main factor explaining the loss of grip force with wrist angle variations. This phenomenon seems to be the results of changes from all muscles responsible for the wrist equilibrium. These findings open the possibility of discovering new criteria indicating how wrist posture is controlled during grasping tasks. For instance, a predictive model could be developed to predict the optimum wrist position for grip force production based on the minimisation or maximisation of a criterion related to the length of the muscles. Figure 4 presents an exploratory visualisation of the data from our experiment to find such a criterion. It presents the sum over all four muscles of the distance between their current length and their optimum length (Eq. (1)) against wrist angle. This figure strongly suggests that the spontaneous wrist angle, i.e. the optimum position, seems to correspond to the minimum of this sum. This minimum sum can be understood as the fact that optimum posture is the result of the most optimum configuration for all four muscles, in terms of the force-length relationship. Thus, the sum of the lengthening from all the muscles seems to represent a parameter that could be used to predict the optimum wrist angle position. Developing and testing models based on such criterion could help to understand the complex couplings existing between joint position, muscle force and performance in a task by linking an observed behaviour (optimum wrist position) and the state of the neuromuscular system (muscle length and force capacities). Further studies should focus on other grasping tasks and force levels to clarify whether this criterion can be generalised to all hand force production tasks or different patterns emerge depending on the constraints of the task.

1 $$\Delta {L}_{norm}=\mathop{\sum }\limits_{i=1}^{4}|\frac{{L}_{opt}^{i}-{L}_{\Theta }^{i}}{{L}_{opt}^{i}}|$$

However, this study presents some limitations. First, the wrist geometrical model used in the present study is based on corpse data and the use of a double-cylinder wrapping is a simplification of its complicated musculoskeletal system. To our knowledge, no other model represents more accurately the tendon kinematics at the wrist. Further studies could be engaged with *in vivo* imagery techniques to develop models that provide a more physiologically realistic estimation of the tendon excursion at the wrist. Secondly, we focused on a specific grip posture and a maximum voluntary force task, which represents a small part all hand movement.Further studies should focus on other types of grasping and sub-maximum levels of force production, to investigate whether the relationships between joint postures, muscle capacities and performance observed here are still valid for other biomechanical configurations and/or task requirements. Finally, regression analyses gave a low correlation coefficient. Although several factors could explain those poor correlations, such as joint-angle dependency on neuromuscular properties^23^, the main reason was probably that only four muscles were considered against a total of 30 to actuate the five fingers and the wrist. Adding more muscles in the musculoskeletal model, for instance by considering the wrist ulnar deviators, deep flexors or individual muscles for each finger could provide more accurate results, e.g. a higher correlation coefficient, but would require more experiments to determine their force-length relationship according to the protocol developed recently^19^. Nevertheless, the goal of these analyses was to compare how different combinations of muscles could explain grip force variations, and they already have answered our hypothesis by showing that the force or length of FDS alone cannot explain the grip force variations associated with wrist posture, while a model with more muscles gives better results.

To conclude, thanks to both the elaborate grip protocol and a hand musculoskeletal model this is the first time in the field of hand biomechanics that data have been provided to study the link between external performance and the muscles’ physiological state. More precisely, this approach allows quantifying the influence of wrist angle on muscle capacities by taking into account the finger and wrist postures as well as electromyography. The data obtained are relevant for both the understanding of motor control and joint abundance as well as for handling ergonomics. From the ergonomic point of view, the shape and dimensions of handles and the constraints of manual tasks in the workplace could be adapted to prevent musculoskeletal disorders associated with awkward wrist postures, such as carpal tunnel syndrome or lateral epicondylitis^24^. On a fundamental level, our results suggest that the force-generating capacities may play a primary role in how the neuromusculoskeletal system is organising when facing the muscle and joint abundances. The results from this study illustrate how biomechanical models can provide relevant information for motor control and behavioural neuroscience approaches. For instance, force and length values estimated by the model could help to investigate motor control theories relying on muscle properties, such as the equilibrium point hypothesis^25^, or to explain patterns of muscle synergies observed via EMG signals, e.g. whether they emerge from neural commands or biomechanical constraints^26^.

## Methods

### Participants

Sixteen volunteers (8 men & 8 women) took part in the study. Their anthropometric data are detailed in Table 2. None of them had known trauma or neuropathy in the hand or upper extremity in the six months that preceded the experiment. Only participants whose hands were longer than 17 cm were included. In this way, the chosen diameters of the handle matched with 15% to 25% of participants’ hands. Before starting the experiment, anthropometric measurements were taken on each participant and included both hand and wrist dimensions and muscle lengths (Table 2). The protocol was approved by the Aix-Marseille ethics committee. Experiments were conducted in accordance with the guidelines of the ethics committee and all participants gave their informed consent.

**Table 2:** Mean anthropometric data of the participants in cm and age in years.

	Mean	SD
Age	21, 7	2, 5
Height	170, 4	6, 9
Lhand	18, 4	1, 0
*L*0_*EDC*_	41, 4	3, 5
*L*0_*EDR*_	32, 4	3, 7
*L*0_*FCR*_	33, 9	3, 5
*L*0_*FDS*_	39, 1	4, 8

### Experimental procedure

Participants were asked to exert their maximum grip force on a cylindrical handle. They were standing with the shoulder at about 30 degrees of flexion and adduction, and elbow at 45 degrees of flexion. They grasped the handle with the four long fingers (index, middle, ring and little), in an adapted power grip posture^27^, to avoid different finger force sharing induced by variations of thumb positions between different diameters.

Three diameters (28 mm, 38 mm, and 48 mm) were tested. These diameters were chosen to cover a range centred on a mean optimum size defined in the literature^12^. For each diameter, maximum grip force was exerted in four different wrist postures. Three postures were prescribed: maximum flexion (F), maximum extension (E) and neutral (N) posture, which were considered as the alignment of the longitudinal axes of the third metacarpal and radius (Wrist at 0 degrees of flexion and adduction). For the last posture, referred to as “spontaneous” (S), participants were instructed to choose the most appropriate position to produce maximum force. This was made possible by the degree of freedom of the mechanical arm. The maximum flexion and extension were considered as the extreme position in which the participant was still able to exert maximum grip force comfortably.

Each condition was repeated twice, resulting in 24 trials by participant (4 postures * 3 diameters * 2 repetitions). The order of the diameters was randomised to avoid the effect of muscle fatigue. Regarding the order of the postures, the spontaneous (S) posture was first tested, and the order of the other trials was randomised. A rest period of at least 2 min 30 and 10 min was respected between two trials and two diameters, respectively. For each combination of posture and diameter, only the trial corresponding to the maximum grip force between the two repetitions was used for data processing.

### Material

**Figure 5:**
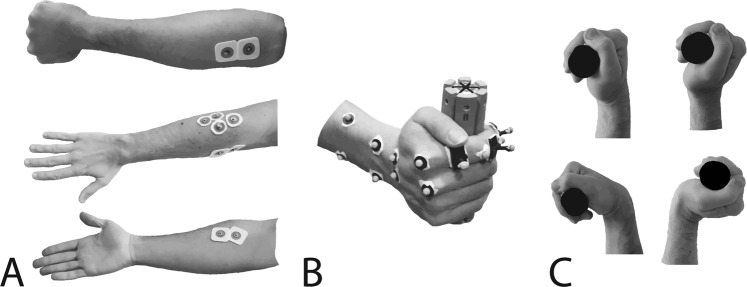
(**A**) Placement of the electrodes for, from top to bottom, FDS, EDC, ECR and FCR. (**B**) Placement of the markers and fingers’ posture on the handle. (**C**) Figure of the four positions, from top left to bottom right, Neutral (N), Spontaneous (S), Flexion (F) and Extension (E).

A motion capture system with seven cameras (Vicon, Oxford, UK; freq: 100 Hz) was used to track the movements of 14 markers placed on the dorsal aspects of the wrist and index finger segments. The placement of these markers corresponded to anatomical landmarks of the hands and was adapted from literature recommendations^28^. Eleven hemispherical markers with a diameter of 6 mm were placed on anatomical landmarks of the hand and three 5-mm deported markers, one for each phalanx, were fixed on the index finger Fig. 5.

Grip force was recorded with an instrumented handle (Sixaxes, Argenteuil, FR)^12,29^ at 1000 Hz. This handle is divided into 6 beams, each equipped with strain gauge sensors. The diameter of the handle could be changed by screwing resin plates of various sizes resulting in handle diameters of 28 mm, 38 mm and 48 mm. The handle was mounted on a mechanical arm. Using the arm, the position and orientation of the handle could be adjusted to the participant’s anthropometry. Once its position was adjusted, the handle could still move slightly so that the participant could exert a grip force on the handle without lifting the object or applying any other force, such as pulling pushing or torque application. The handle could also rotate slightly around its main axis to help the participant adopting a wrist position.

Electromyographic signals were acquired with a wired Biopac system (MP-150, BIOPAC Systems, Inc., Goleta, CA, USA). The signals of four major muscles were recorded: flexor carpi radialis (FCR), flexor digitorum superficialis (FDS), extensor carpi radialis (ECR), extensor digitorum communis (EDC). The recording was performed at a frequency of 1000 Hz and synchronised with grip force and kinematics using the Vicon system. Before placing electrodes, the skin was shaved, sanded and rinsed with an alcoholic solution. The placement of the electrodes followed a previous study^30^. Subjects also performed functional tasks^30,31^, facilitating the activation of each muscle, to verify correct placement of the electrodes to avoid cross-talk issues.

### Data processing

The index finger and wrist joint angles were estimated from kinematic data. First, the marker positions were used to compute the distal and proximal segment coordinate systems and the relative rotation matrix. These coordinate systems were calculated from the positions of 3 markers for each segment. The longitudinal (X) axis was calculated from the unit vector of the distal to the proximal marker vector. The sagittal (Z) axis was orthogonal to the plane that includes the X and the third segment marker. The Y-axis is the cross product of X and Z. Then, the joint angles were extracted from the rotation matrix using a Z-Y-X (flexion/abduction/pronation) sequence of Cardan angles. Two degrees of freedom, in flexion/extension and adduction/abduction, were considered for the wrist and index finger metacarpophalangeal joints. Interphalangeal joints were considered as hinge joints, with only one degree of freedom in flexion/extension.

The signal recorded at each beam of the handle was first filtered (Butterworth low pass zero-phase filter at 5 Hz, order 2). The grip force was then calculated as the sum of the six forces recorded by the handle. Maximum grip force corresponded to the mean of the grip force on a 500 ms window analysis centred on the maximum force peak. For each participant, the normalised maximum grip force (MGF) was calculated as the ratio between the maximum grip force determined in the trial and the maximum value recorded for that participant among all trials.

On the same analysis windows, EMG signals were filtered with a bandwidth Butterworth (20–400 Hz, order 4). Root Mean Square (RMS) was calculated. For each muscle, the muscle activation was calculated by normalising the RMS value in a trial by the maximum RMS value in all trials recorded for the participant.

### Musculoskeletal model

**Figure 6:**
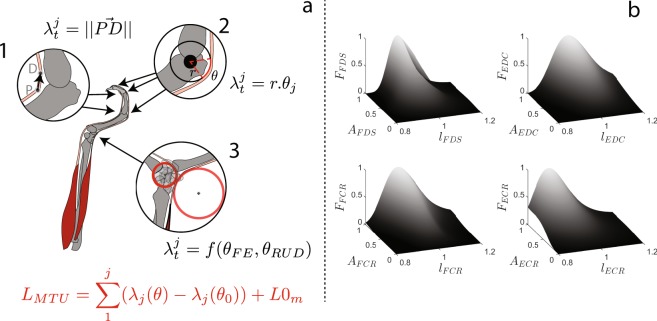
Representation of the geometrical models developed that calculate the muscle-tendon unit (*L*_*MTU*_) as the sum of tendon excursion obtained from each joint model. ${\lambda }_{t}^{j}$ is the different tendon segment length at each joint. *θ*_*j*_ is the joint angle captured with the markers. *r* is the radius of the circle where tendons wrap (Model 2). *L*0_*m*_ is the anthropometric length measured on participants at the neutral position. P and D are a proximal and distal point, respectively, of the tendon at a joint (Model 1).

A musculoskeletal model was used to compute the current length and force of each of the four muscles using the experimental data of joint angles, grip force and EMG as input. The model is divided into two steps. The first steps consists in using a geometric model (Fig. 6) to calculate the muscle-tendon unit (MTU) length from the experimental angles and anthropometric measurements, and the second consists in using a force-length-activation model to calculate muscle length and force from the calculated MTU length and measured activation.

#### Musculo-tendon excursion

For the first step, the musculo-tendon excursion (i.e. lengthening or shortening of the tendon at each joint from the neutral posture) is estimated at each joint. Estimations at the index finger (DIP, PIP and MCP) joints were done using anatomical data^32^ and geometric models^33^ with different models for the two extrinsic muscles, i.e. EDC and FDS. The EDC tendon excursion is calculated based on a constant moment arm model as the product of the moment arm (radius) and the joint angle (Landsmeer Model I^33^, Fig. 6, a.2). The FDS tendon excursion was estimated according to a bow-string model^32^ as the variation of the distance between a proximal and a distal point representing the path of the tendon at the joint (Fig. 6, a.1). At the wrist joint, the excursion of each of the four muscles (FCR, ECR, FDS, EDC) was estimated using anatomical data and a geometrical model based on a cylindrical wrapping method^21,34^, using two cylinders, one for wrist flexion positions and one for wrist extension (Fig. 6, a.3). Parameters of the two cylinders were determined for each muscle using an optimisation procedure described in Supplementary Information. For each muscle, an MTU lengthening is then calculated by summing the excursions of the tendon estimated at the four joints. The current MTU length was finally determined as the sum of the MTU lengthening and the MTU length at the neutral position, measured for each subject as detailed in Hauraix *et al*.^19^.

#### Muscle length and force

The second part of the model consisted in calculating the normalised muscle length and muscle force from the MTU length and EMG activation using the force-length-activation relationships developed by Hauraix, *et al*., for flexors (FCR and FDS)^19^ and extensors (ECR and EDC, unpublished). Briefly, the model estimates the muscle length *l*_*m*_ from the MTU length (*L*_*MTU*_) at each trial and the MTU length at the reference position (*L*0_*m*_) at given activation levels. Muscle force was computed using activation levels and *l*_*m*_. The quadratic relationships used were the ones describing the mean relationship for all subjects (see average model in the article).

### Statistical analysis

The analysis was made with Statistica software (TIBCO Software Inc., CA, USA). For the experimental angle and grip force, a two-way repeated ANOVA comparing the effect of *posture* and *diameter* against the different independent variable (Wrist angle or Normalised MGF) was conducted. Three-way repeated ANOVA models were conducted to analyse the effect of *posture*, *diameter* and *muscle* on muscle activation, muscle length or muscle force. For each ANOVA, Tukey HSD post hoc analyses were used to evaluate differences between conditions. Significance level was fixed at *p* = 0.05.

Multiple regression analyses were conducted in order to determine possible correlations between MGF and either muscle forces or lengths. For each possible combination of muscles, four regressions or multiple regressions were tested using either a linear or a quadratic model based on either muscle force or muscle length. For each combination, the Akaike Information Criterion (AIC) was computed to select the model that best fits the MGF experimental data. To compare the different models, Δ_*AIC*_ was calculated and the most plausible models^35^ determined. Models with a value less than two are considered substantial, a value between 2 and 4 are considerably less plausible, and models above that essentially implausible.

## Supplementary information

Complex couplings between joints, muscles and performance the role of the wrist in grasping

## Data Availability

The datasets generated and/or analysed during the current study are available from the corresponding author on reasonable request.
