# Supplementary material for: Complex couplings between joints, muscles and performance: the role of the wrist in grasping
Source: Sci Rep. 2019 Dec 18;9:19357. doi: 10.1038/s41598-019-55443-w (PMC6920170; doi:10.1038/s41598-019-55443-w)
Supplement: Supplementary file 1 — Complex couplings between joints, muscles and performance the role of the wrist in grasping [file 41598_2019_55443_MOESM1_ESM.pdf]

Complex couplings between joints, muscles and performance the role of the wrist in grasping

Mathieu Caumes[1,*], Benjamin Goislard De Monsabert[1,], Hugo Hauraix[1], Eric Berton[1], and Laurent Vigouroux[1]**

[1]Institute of Movement Sciences, National Centre for Scientific Research, Aix-Marseille University, Marseille, 13009, France
[*]mathieu.caumes@univ-amu.fr
[**]benjamin.goislard-de-monsabert@univ-amu.fr

Supplementary Information

This document provides details on the geometric model used to evaluate musculotendon length used in the study "Complex couplings between joint, muscles and performance: the role of the wrist in grasping" by Caumes et al.

Double-cylinder geometric Model

The geometrical model describing the musculo-tendon kinematics concerning the wrist joint is based on a cylindrical wrapping model [1]. Considering the wide range of motion of the wrist and the fact that tendons alternatively wrap on different anatomical structures, two cylinders were used to model the path of each tendon. One of them describes the wrapping around the wrists bones on flexion or extension for extensors or flexors, respectively. The other cylinder describes the wrapping around soft tissues in the opposite direction. These cylinders are defined by three parameters describing their position from the joint's centre (anteroposterior, X, and longitudinal, Y, position and their radius).

Determination of cylinder parameters

Parameters provided in the literature were determined manually and did not concern extrinsic muscles [2].To improve on this earlier study and obtain data for all four muscles studied here, an optimisation procedure was used to determine the cylinder parameters for the four muscles. The procedure aimed at finding the cylinders that reflect the best anatomical and functional reality of the wrist. More precisely, on a whole ranges of motions, the tendon could either wrap on one cylinders or none. The moment arms estimated with the cylinders obtained had to be consistent with curves available in the literature[3, 4]. Adjustment of the parameters was done using a RMS deviation constrained optimisation (Fmincon, optimisation toolbox, Matlab, Natick, Massachusetts USA) with upper and lower bounds formulated bellow. The optimisation process was executed for each muscle as follows :

Find

$$P = \begin{bmatrix} X_{c1} & X_{c2} \\ Y_{c1} & Y_{c2} \\ R_{c1} & R_{c2} \end{bmatrix} \tag{1}$$

that minimise

$$f(P) = \sqrt{\frac{\sum_{\theta=-60}^{60}(MA_{Model}(\theta) - MA_{Litt}(\theta))^2}{n}} \tag{2}$$

subject to

$$\begin{cases} 0 < \alpha < C1_i \\ -C2_i < \beta < C2_i \end{cases} \tag{3}$$

where X_{cn}, Y_{cn}, are the coordinates describing the position of the cylinder axis along anteroposterior and longitudinal axes, respectively, and R_{cn} being its radius. MA_{Model} is the normalised moment arm of the musculo-tendon unit at centre of the wrist joint and calculated by the wrapping model. MA_{litt} is the normalised moment arm data taken from a polynomial equation in the literature[3, 4]. Normalisation consisted in subtracting the mean moment arm of the dataset and divided by its range of value. This normalisation was conducted to avoid absolute differences between moment arm values due to the anatomical specification (wrist width principally) of the arm on which the studies defines the moment arm. n is number of wrist FE angle, θ, values tested on the whole range of motion. α is the angle between vectors tangent of cylinders originating from the proximal point of tendon P. $C1_i$ and $C2_i$ are constraint values for the different muscles (see in Table S1). Optimisation boundaries, starting points and constraints values were determined in order to get plausible anatomical and functional geometries and moment arm. Parameters found for each muscles are presented in Supplementary Materials Table S1.

Muscle	C1	C2	X_{c1}	Y_{c1}	$R_{c1}(mm)$	X_{c2}	Y_{c2}	$R_{c2}(mm)$	$f(P)(mm)$
ECR	10	5	-13.08	25.77	15	-5.88	1.57	6	0.04
EDC	2		-9.98	14.27	3	3.59	1.02	13.39	0.07
FCR	1	10	3	-5.6	15	25	1.17	5.1	0.26
FDS	5	1	2.38	2.39	12.34	22.58	11.26	2.66	0.19

Table S1. Parameters for each cylinder in flexion and extension for the four muscles.

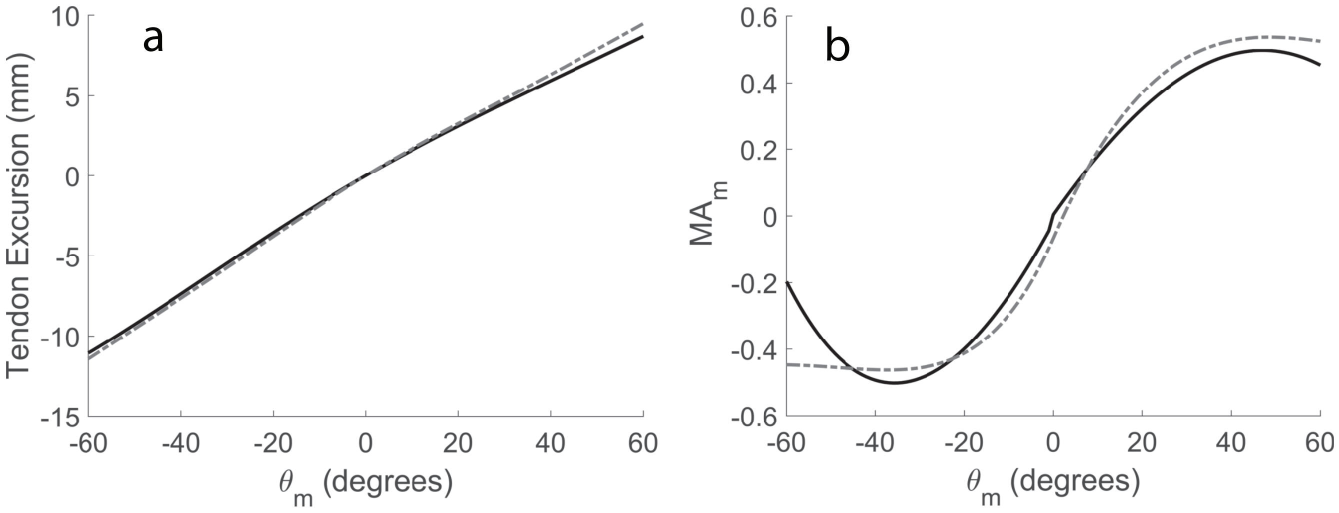

Figure S1. *a*. ECR tendon excursion in mm against the wrist range of motion in degrees. *b*. Moment arm of the ECR in cm against wrist range of motion. Solid line represents the optimisation results, dotted line the results from the literature polynomial [3].

References

1. Charlton, I. W. & Johnson, G. R. Application of spherical and cylindrical wrapping algorithms in a musculoskeletal model of the upper limb. *J. Biomech.* **34**, 1209–1216, DOI: https://doi.org/10.1016/S0021-9290(01)00074-4 (2001).
2. Goislard de Monsabert, B. G. D., Edwards, D., Shah, D. & Kedgley, A. Importance of Consistent Datasets in Musculoskeletal Modelling: A Study of the Hand and Wrist. *Annals Biomed. Eng.* **46**, 71–85, DOI: https://doi.org/10.1007/s10439-017-1936-z (2018).
3. Paclet, F. *Analyse biomécanique des transferts tendineux de la main (technique Tsugé) Modélisation des tensions Suivi longitudinal des patients*. Ph.D. thesis, Université de Grenoble (2010).
4. Lemay, M. A. & Crago, P. E. A dynamic model for simulating movements of the elbow, forearm, and wrist. *J. Biomech.* **29**, 1319–1330, DOI: https://doi.org/10.1016/0021-9290(96)00026-7 (1996).
